# The combined expression of the stromal markers fibronectin and SPARC improves the prediction of survival in diffuse large B-cell lymphoma

**DOI:** 10.1186/2162-3619-2-27

**Published:** 2013-10-08

**Authors:** Simone Brandt, Chiara Montagna, Antoin Georgis, Peter J Schüffler, Marco M Bühler, Burkhardt Seifert, Thore Thiesler, Alessandra Curioni-Fontecedro, Ivan Hegyi, Silvia Dehler, Vittoria Martin, Marianne Tinguely, Davide Soldini

**Affiliations:** 1 Institute of Surgical Pathology, University Hospital Zurich, Zurich, Switzerland; 2 Department of Computer Science, ETH Zurich, Zurich, Switzerland; 3 Division of Biostatistics, Institute for Social and Preventive Medicine, University of Zurich, Zurich, Switzerland; 4 Institute of Pathology, University of Bonn, Bonn, Germany; 5 Department of Oncology, University Hospital Zurich, Zurich, Switzerland; 6 Cancer Registry, Institute of Surgical Pathology, University Hospital Zurich, Zurich, Switzerland; 7 Institute of Pathology, Locarno, Switzerland; 8 Kempf and Pfaltz, Histologische Diagnostik, Zurich 8042, Switzerland

**Keywords:** SPARC, Fibronectin, DLBCL, Stromal signature, Extracellular matrix, Tumour microenvironment

## Abstract

**Background:**

In diffuse large B-cell lymphomas, gene expression profiling studies attributed a major biologic role to non-neoplastic cells of the tumour microenvironment as its composition and characteristics were shown to predict survival. In particular, the expression of selected genes encoding components of the extracellular matrix was reported to be associated with clinical outcome. Nevertheless, the translation of these data into robust, routinely applicable immunohistochemical markers is still warranted. Therefore, in this study, we analysed the combination of the expression of the extracellular matrix components Fibronectin and SPARC on formalin-fixed paraffin embedded tissue derived from 173 patients with DLBCL in order to recapitulate gene expression profiling data.

**Results:**

The expression of Fibronectin and SPARC was detected in 77/173 (44.5%) and 125/173 (72.3%) cases, respectively, and 55/173 (31.8%) cases were double positive. Patients with lymphomas expressing Fibronectin showed significantly longer overall survival when compared to negative ones (6.3 versus 3.6 years). Moreover, patients with double positive lymphomas also presented with significantly longer overall survival when compared with the remaining cases (11.6 versus 3.6 years) and this combined expression of both markers results in a better association with overall survival data than the expression of SPARC or Fibronectin taken separately (Hazard ratio 0.41, 95% confidence interval 0.17 to 0.95, p = 0.037). Finally, neither Fibronectin nor SPARC expression was associated with any of the collected clinico-pathological parameters.

**Conclusions:**

The combined immunohistochemical assessment of Fibronectin and SPARC, two components of the extracellular matrix, represents an important tool for the prediction of survival in diffuse large B-cell lymphomas. Our study suggests that translation of gene expression profiling data on tumour microenvironment into routinely applicable immunohistochemical markers is a useful approach for a further characterization of this heterogeneous type of lymphoma.

## Background

Diffuse large B-cell lymphoma (DLBCL) accounts for approximately 25-30% of non-Hodgkin lymphomas in adults in the western countries and includes biologically and clinically heterogeneous subgroups of tumours, which present with distinctive outcomes [1]. The gene expression profiling (GEP) of primary tumour samples led to the identification of two major DLBCL subtypes based on the similarity with maturation stages of B-cell differentiation: those derived from activated B-cells (non-GCB) or germinal-centre B-cell-like cells (GCB) [2,3]. In addition to the properties of the lymphoma cells, the composition of the tumour microenvironment and genes expressed by non-tumour cells have also been shown to be useful for the characterization of DLBCL. To this regard, the DLBCL subgroup expressing *de novo* CD5, which represents approximately 10% of all DLBCL cases and carries a more aggressive behaviour, presents with down-regulation of genes associated with extracellular matrix (ECM), such as SPARC (secreted protein acidic and rich in cysteine) [4-6]. Moreover, stromal gene signatures, which predicted survival in patients treated with CHOP and R-CHOP, were identified in DLBCL through the separate analysis of the neoplastic and non-neoplastic subpopulation by means of flow cytometry. In particular, a so-called stromal-1 signature reflecting ECM deposition and histiocytic infiltration was associated with better clinical outcome [7]. *Fibronectin* (FN1) and *SPARC* were among the genes included in this stromal-1 signature. FN1 is a 250-kDA glycoprotein composed of 2 similar polypeptides [8] and can present as soluble plasma dimer, mainly released by hepatocytes, or as an insoluble multimer, secreted by different cell types, such as epithelial cells, fibroblasts and macrophages [9]. In the latter form, FN1 represents a major component of the ECM and through its different domains it can interact with other proteins of the ECM, glycosaminoglicanes, as well as cell receptors, in particular integrins [8]. SPARC, also called osteonectin, is a highly conserved 43-kDa glycoprotein which can be expressed by osteoblasts, endothelial cells, fibroblasts, macrophages, as well as by a variety of tumour cell types. It functions by regulating cell adhesion, ECM remodelling, and growth factor signalling and has been shown both to induce tumour progression and suppress tumour growth, depending on the tumour type [10]. Whereas the protein expression of SPARC was shown to correlate with better prognosis in DLBCL [7,11], the one of FN1 has not been studied so far.

The translation of GEP data onto the protein level by means of immunohistochemistry (IHC) represents an important step for routine diagnostic purposes. For this reason, in the present study we analysed the IHC expression of both FN1 and SPARC in relationship with clinic-pathological data in a large series of DLBCL on tissue microarrays (TMA).

## Results

### Characteristics of patients and tumours

Our cohort of 173 patients included 94 (54.3%) men and 79 (45.7%) women. Patients ranged in age from 15 to 95 years, with a median age of 64 years. Of these, 67 (38.7%) were younger than 60 years and 106 (61.3%) were older than 60 years. The median follow-up for the 173 patients was 5.4 years, ranging from 0.02 to 19.35 years. Based on IHC, 67 (38.7%) cases were classified as GCB and 106 (61.3%) as non-GCB, according to the Hans algorithm. CD5 expression was detected in 6 out of 173 (3.5%) cases. By means of fluorescence *in situ* hybridization (FISH) analysis, translocations for BCL2, BCL6 and MYC were detected in 23 (13.3%), 31 (17.9%) and 6 (3.5%) cases, respectively. Summary of the characteristics of tumours and patients are presented in Tables 1 and 2.

**Table 1:** Clinical characteristics of the patients

**Clinical information**		**Number (%)**
Age at diagnosis	Mean	61.6
	Median	64
	<60 years	67 (38.7)
	>60 years	106 (61.3)
Gender	Female	79 (45.7)
	Male	94 (54.3)
Localisation	Nodal only	79 (45.7)
	Extranodal only	61 (35.3)
	Nodal and extranodal	30 (17.3)
	No data available	3 (1.7)
Stage	I	26 (15.0)
	II	21 (12.1)
	III	16 (9.2)
	IV	16 (9.2)
	No data available	94 (54.3)
Chemotherapy	CHOP-like regimen	72 (92.5)
	Intensive regimen	2 (1.2)
	VACOP	3 (1.7)
	Low-intensity regimen	6 (3.5)
	No treatment	2 (1.2)
Rituximab	Yes	49 (28.3)
	No	43 (24.9)
	No data available	81 (46.8)
Rituximab (patients with CHOP-like regimen)	Yes	45 (26.0)
	No	27 (15.6)
	No data available	101 (58.4)
Radiotherapy	Yes	24 (13.9)
	No	59 (34.1)
	No data available	90 (52)
Response to treatment	Complete remission	49 (28.3)
	Recurrence	47 (27.2)
	Death without disease-free interval	41 (23.7)
	Loss of follow-up	36 (20.8)

**Table 2:** Immunohistochemical and genetic characteristics of the lymphomas

**Immunohistochemical analysis**		**Number (%)**
CD10 expression	Positive	49 (28.3)
	Negative	124 (71.7)
BCL6 expression	Positive	59 (34.1)
	Negative	114 (65.9)
MUM1 expression	Positive	44 (25.4)
	Negative	129 (74.6)
GCB/non-GCB phenotype	GCB	67 (38.7)
	non-GCB	106 (61.3)
Fibronectin expression	High	77 (44.5)
	Low	96 (55.5)
SPARC expression	High	125 (72.3)
	Low	48 (27.7)
CD5 expression	Positive (>20%)	6 (3.5)
	Negative (<20%)	167 (96.5)
**FISH analysis**		
Number of translocations	0	116 (67.1)
	1	53 (30.6)
	2	4 (2.3)
FISH for BCL2	translocation	23 (13.3)
	no translocation	131 (75.7)
	not evaluable	19 (11)
FISH for BCL6	translocation	31 (17.9)
	no translocation	55 (31.8)
	not evaluable	87 (50.3)
FISH for MYC	translocation	6 (3.5)
	no translocation	117 (67.6)
	not evaluable	50 (28.9)

### Expression of fibronectin and SPARC in DLBCL

**Figure 1:**
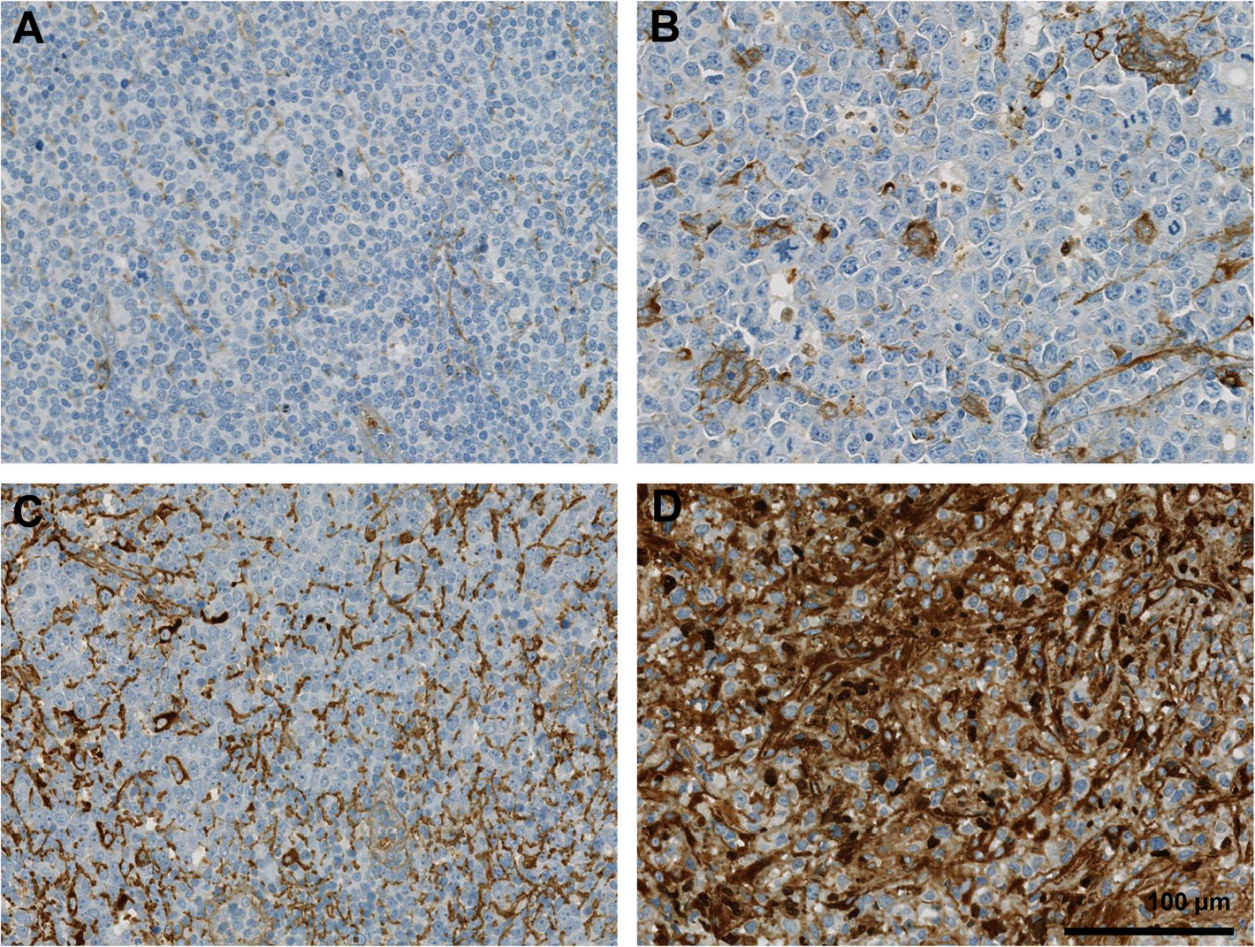
**Fibronectin staining highlights fibrous strands of the ECM between tumour cells of DLBCL.** Four DLBCL cases with different staining intensities are shown. **A**. and **B**. DLBCL cases scored 1 and 2, respectively, and considered as FN1-low. **C**. and **D**. DLBCL cases scored 3 and 4, respectively, and considered as FN1-high.

**Figure 2:**
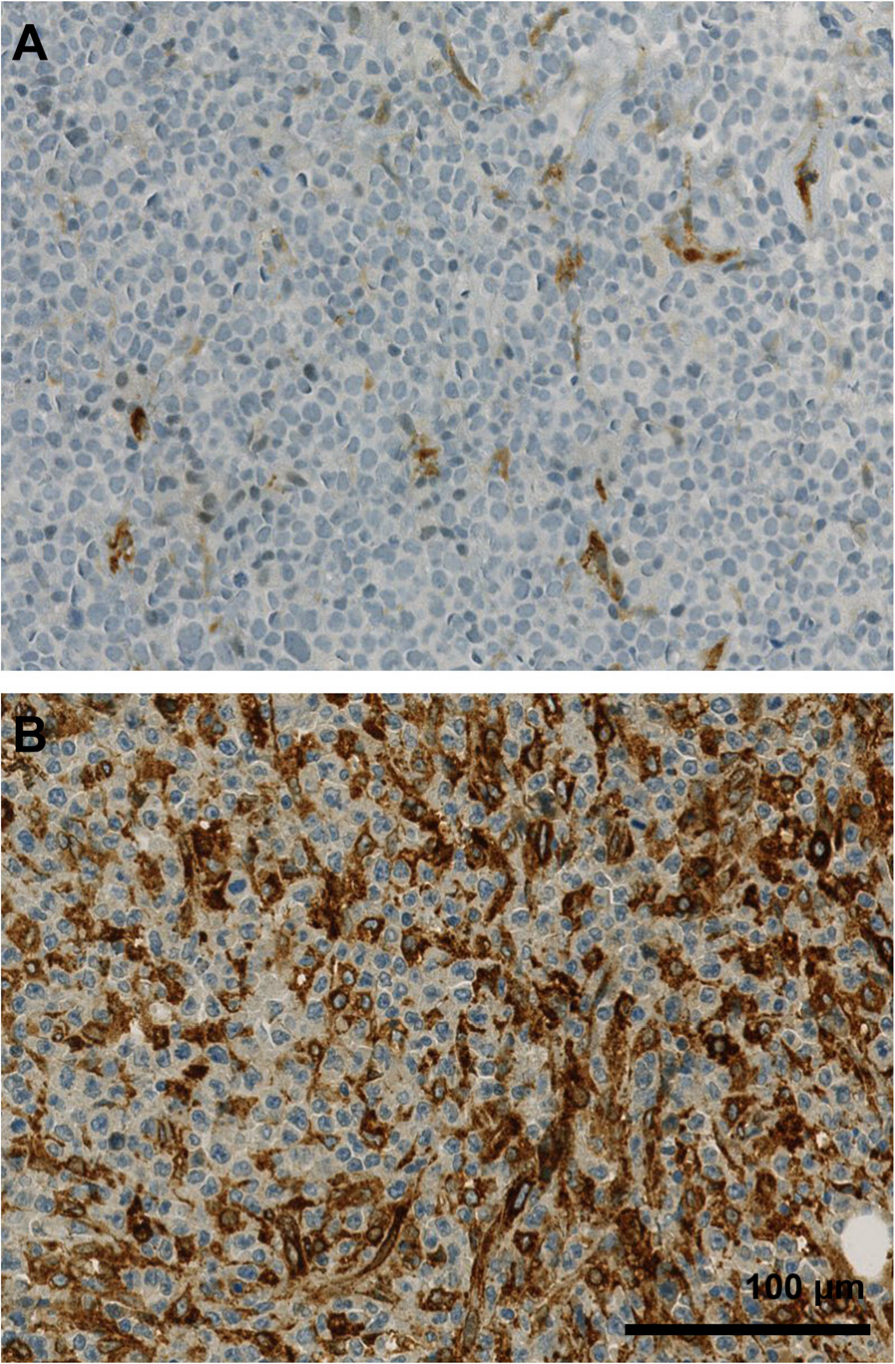
**SPARC staining was detected primarily in non-tumour cells and in endothelial cells of blood vessels.** Blood vessels were not considered in our analysis. A DLBCL case showing merely scattered positive cells apart from few positive blood vessels and scored negative for SPARC (SPARC-low) **(A)**. A DLBCL case with a high number of SPARC positive cells of the microenvironment and considered as SPARC-high **(B)**.

FN1 staining highlighted fibrous strands of the ECM between tumour cells and 96 cases (55.5%) resulted as FN1-low, whereas 77 cases (44.5%) as FN1-high (Figure 1). SPARC expression was observed in non-neoplastic cells. Out of the 173 cases, 48 (27.7%) were considered as SPARC-low, whereas 125 cases (72.3%) contained at least 10% positive non-neoplastic cells and were classified as SPARC-high (Figure 2). The results for the combined expression of FN1 and SPARC showed: 55 (31.8%) cases were FN1-high/SPARC-high, 22 (12.7%) FN1-high/SPARC-low, 70 (40.5%) FN1-low/SPARC-high, and 26 (15.0%) FN1-low/SPARC-low.

In a Pearson’s chi-squared test, we could not find any significant association between the expression of SPARC and of FN1 (p-value = 0.19). Moreover, no significant association was found between the expression of either ECM markers and the GCB or non-GCB phenotype, CD5 expression, molecular aberrations for BCL2, BCL6 and MYC or the collected clinico-pathological parameters (gender, tumour site, and tumour stage).

### The combined expression of FN1 and SPARC correlates with longer overall survival (OS)

In a Kaplan-Meier analysis of OS, patients with FN1-high expression showed a significant longer OS compared with patients presenting with FN1-low expression (median survival time of 6.3 years for FN1-high cases *versus* 3.6 years for FN1-low cases, p-value = 0.05). Regarding SPARC expression, SPARC-high cases showed longer OS, even though this association was not significant (median survival 5.0 years for SPARC-high cases *versus* 2.5 years for SPARC-low cases, p-value = 0.083). In contrast, expression of neither FN1 nor SPARC was associated with longer progression free survival (PFS) (p-value = 0.8 and p-value = 0.2, respectively).

**Figure 3:**
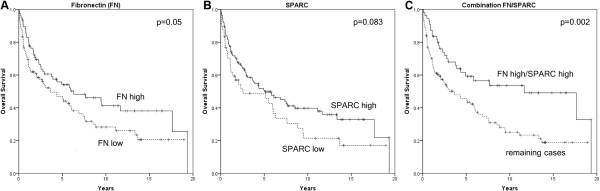
Kaplan-Meier analysis showing overall survival of 173 DLBCL cases separated on the basis of their expression of fibronectin (A), p = 0.05, SPARC (B), p = 0.083 and combined FN1 high/SPARC high expression (C), p = 0.002.

Moreover, double positive SPARC-high/FN1-high cases presented with a significantly longer OS when compared with the remaining ones (median survival time of 11.6 years *versus* 3.6 years, p-value = 0.002) (Figure 3). A Cox-regression analysis showed that the combination FN1-high/SPARC-high had a significantly longer OS when adjusted for the SPARC-high and FN1-high expressions (Hazard ratio 0.41, 95% confidence interval 0.17 to 0.95, p = 0.037).

## Discussion

In previous studies, the expression of the protein SPARC was shown to predict longer OS [7,11,12]. Nevertheless, the IHC expression on formalin-fixed paraffin embedded tissues of other ECM components, such as FN1 or their combination, has not been investigated so far. In the present study, we show that DLBCL cases with expression of FN1 present with longer OS. Moreover, in our cohort the combined expression of FN1 and SPARC is a better predictor for longer OS than the markers taken separately. Since our study comprises mostly cases diagnosed before the introduction of Rituximab as treatment option, only 49 patients received this drug in our cohort. When the analysis for OS was performed in this subgroup of patients no significant difference based on SPARC, FN1 or the combination of both markers was found, possibly due to the low number of cases (data not shown).

Our results, generated by IHC, recapitulate data from a GEP study on a large number of DLBCL. The authors identified several genes of the ECM, and grouped them into so-called stromal signatures, able to predict survival [7]. Stromal-2 signature genes included markers for endothelial cells and key regulator for angiogenesis. In contrast, stromal-1 signature encoded components of the ECM, such as various collagen and laminin isoforms, as well as FN1 and SPARC. In accordance with its role in ECM formation, staining for FN1 localized to the fibrous strands in our lymphomas. SPARC staining, instead, highlighted endothelial cells and non-neoplastic cells, which were identified as macrophages in previous studies, based on CD68 expression [7,11]. Even though several data suggest that the composition of ECM is of major importance in the tumourigenesis, the exact functions of SPARC and FN1 in DLBCL have not been studied so far. To this regard, the role of SPARC could be related to its regulatory activity in several signalling pathways, such as TGFβ and PI3K/AKT. Moreover, SPARC has been shown to bind to FN1 fragments and to induce matrix metalloproteinases (MMP) which degrade ECM components [10].

Our results, together with data derived from studies on tumour microenvironment performed in other tumour types, might open new diagnostic and therapeutic possibilities. To this regard, both FN1 and SPARC are being tested as biomarkers in different clinical trials (see http://clinicaltrials.gov, identifiers: NCT01288963, NCT01442974, NCT01566435). In addition, new therapeutic strategies targeting the ECM components are being evaluated, such as specific molecules inhibiting the interaction between FN1 and tumour cells or therapeutic agents conjugated to antibodies specific for tumour environment. For example, interleukin-2 has been conjugated to an antibody specific for the FN1 isoform containing the extradomain-B, EDB, which is expressed during tissue remodelling in tumours [13,14].

In conclusion, our results show that the combined IHC expression of FN1 and SPARC can be used in the routine clinical practice as predictor of survival in patients suffering from DLBCL, recapitulating the data derived from GEP studies. This suggests that both the ECM component FN1 and the ECM remodelling SPARC can influence the survival of lymphoma cells and their interactions with the microenvironment.

## Methods and patients

### Case selection and construction of tissue microarray

A series of 173 DLBCL diagnosed between 1990 and 2009 were retrieved from the database of the Institute of Surgical Pathology, University Hospital Zurich (PathoPro software, Institute for medical software, Saarbrücken) and reviewed independently by two pathologists (D.S. and M.T.) following the current WHO classification [1].

Collected clinical data included age, gender, tumour site, and tumour stage. For 85 patients, data on the therapeutic approach were available and consisted of CHOP or CHOP-like regimens (72 patients, 45 of them with Rituximab), intensive regimens (HyperCVAD/MTX-AraC, high-dose MTX or M-ACOD; 2 patients, 1 of them with Rituximab), VACOP (vincristine, adriamycin/doxorubicin, cyclophosphamide, etoposide, and prednisone; 3 patients, 1 of them with Rituximab) or low-intensity regimen (chlorambucil and prednisolone; 6 patients, 2 of them with Rituximab). Two patients received no therapy. Collection of survival data and follow-up was carried out in collaboration with the Cancer Registry Zurich.

A tissue microarray (TMA) containing 2 representative tumour tissue core of 0.6 mm in diameter for each case was constructed using formalin-fixed paraffin embedded tissues, as previously described [15].

This study was in accordance with the Helsinki declaration and Swiss laws and was approved by the official authorities of the ethical committee of the Canton Zurich (StV2-2007).

### Immunohistochemistry and FISH

Staining for lymphoma classifications were carried out as recently published [15]. Additionally, 3-μm-thick sections of the TMA were stained in a Ventana ES instrument (Roche’s Ventana Medical Systems, Basel, Switzerland) for FN1 (polyclonal rabbit anti human A0245, DAKO), and SPARC (monoclonal mouse anti-human AON-5031, Santa-Cruz) and evaluated by two pathologists (S.B. and D.S.). By means of CD20 staining tumour areas were identified and the presence of at least 30% of tumour cells for each core was confirmed. MUM1/IRF4, CD10 and BCL6 stainings were scored as negative or positive with a cut-off at 30% and cases were classified either into GCB or non-GCB subtypes, according to the Hans algorithm, as previously described [16]. For CD5, positivity was considered when more than 20% of tumour cells expressed this marker [5].

Regarding FN1, the staining intensity of fibrous strands of the extracellular matrix in tumour areas was considered [7]. Cases were scored form 1+ (negative to weak expression) to 4+ (very strong expression) and the range was then divided into a FN1-low category (1+ and 2+) and a FN1-high (3+ and 4+) category. For SPARC, the number of positive non-neoplastic cells in tumour areas was graded in 10% increments and cases were classified as SPARC-low if less than 10% of non-neoplastic cells were positive and as SPARC-high if at least 10% of non-neoplastic cells were positive for SPARC, based on reports of previous studies [11]. FN1 and SPARC staining in blood vessels was not considered, as genes related to blood vessels are not included in the stromal-1 signature [7]. FISH analysis for BCL2, BCL6 and MYC were performed using probes with a split signals strategy (DAKO) for all cases on TMA, as previously described [15].

For all statistical analysis each case was averaged over both its TMA cores.

### Statistical analysis

Association of the expression of FN1 and SPARC and the relationship between the expression of the two markers and the clinical parameters, including gender, tumour site, tumour stage, as well as IHC characteristics, such as positivity for CD5 and affiliation to GCB or non-GCB subtype, were evaluated using Pearson’s chi-square tests.

Kaplan-Meier analysis was used to estimate OS and PFS. OS was defined as the time from the lymphoma diagnosis to death of any cause or last contact. PFS was defined as the time from the lymphoma diagnosis to recurrence, death or last contact. The log-rank test was used to evaluate differences in OS and PFS between groups. Cox-regression was used to determine the combined effect of the expression of SPARC and FN1.

Statistical analysis was performed using IBM SPSS Statistics 21 (SPSS Inc, Chicago, IL, USA). P-values ≤0.05 were considered statistically significant.

## Competing interests

The authors declare that they have no competing interests.

## Authors’ contributions

SB, CM, AG, PJS, BS performed the experiments; SB, DS, MT, ACF, TT, SD helped conceive and design the study and interpret the results; SB, DS, MT, IH, MB drafted the manuscript; VM evaluated FISH. All authors read and approved the final manuscript.
